# Coffee Berry Borer (*Hypothenemus hampei*) Emergence from Ground Fruits Across Varying Altitudes and Climate Cycles, and the Effect on Coffee Tree Infestation

**DOI:** 10.1007/s13744-021-00863-5

**Published:** 2021-03-19

**Authors:** Luis Miguel Constantino, Zulma Nancy Gil, Esther Cecilia Montoya, Pablo Benavides

**Affiliations:** 1 Department of Entomology, National Coffee Research Center, Cenicafé, Manizales, Chinchiná, Colombia; 2 Biometrics, National Coffee Research Center, Cenicafé, Manizales, Chinchiná, Colombia

**Keywords:** Coffee berry borer, integrated pest management, *Coffea arabica*, Climate cycles El Niño-La Niña

## Abstract

During coffee harvest, picked berries fall to the ground where they serve as a reservoir for the coffee berry borer (CBB) which then infest coffee berries on the trees. This study tested the effect of fallen CBB-infested coffee berries on the infestation of coffee trees (*Coffea arabica*). Three-year-old trees were treated with either 0, 1, 5, 10, 15, or 20 CBB-infested berries placed on the root vicinity. The CBB infestation of coffee trees was sampled every 30 days during 6 months for four coffee productive cycles. The experiment was set up at four different locations comprising different altitudes (1,218; 1,381; 1,470; and 1,700 m.a.s.l.) and the measurements were taken during 4 years where the climatic events of El Niño, La Niña, Neutral, and transitions El Niño/La Niña were present. The results show that CBB-infested berries left on the ground are a reservoir of CBB for 140 ± 8.2 days and infest developing healthy coffee berries. In a climate Neutral year, one CBB-infested ground berry left on the ground infested on average 590.2 ± 142.2 berries in coffee trees grown at 1,218 m.a.s.l. At the same altitude, one CBB-infested ground berry resulted in 151.5 ± 29.1 infested tree berries during La Niña year and 959.0 ± 89.6 during El Niño year. The CBB infestation was positively correlated with temperature and negatively correlated with altitude (*R*^2^= 0.99 and *R*^2^= −0.96, respectively). This study highlights the importance of careful harvesting practices to prevent berries from falling to the ground, followed by ground sanitation to limit later infestation of the coffee crop.

## Introduction

The coffee berry borer (CBB), *Hypothenemus hampei* (Ferrari) (Coleoptera: Curculionidae: Scolytinae), is the most important pest in all the coffee-producing countries in the world, including Colombia. The adult female CBB bores a hole into the central floral disc of the coffee fruit (referred to as a berry) and waits until the endosperm is >20% dry matter before entering into the seed (referred to as a bean), where she builds galleries for reproduction. The offspring feed on the endosperm tissue, causing direct damage to the bean. The internal feeding habit of this pest makes it particularly difficult to control using chemical pesticides. The CBB takes refuge in the overripe and dried berries that are left on the trees or ground after the harvest season is complete, where they remain until the arrival of the rains the following season, after which they emerge and infest the new coffee crop (Baker 1999; Bustillo 2008; Cenicafé 1995 a,b; Cenicafé 1998; Baker 1999; Benavides et al. 2002; Bustillo 2008; Benavides 2010).

As part of the integrated pest management (IPM) of CBB, timely harvesting practices and the removal of ripe and unripe berries that have fallen to the ground or that remain on the plant are efficient strategies to reduce pest populations in coffee crops and the level of infestation during the next harvest cycle (Bustillo et al. 1998). Based on the conditions of Colombian coffee production, the timely harvest and collection of ripe berries left by collectors are successful and crucial strategies for managing the CBB (Bustillo 2008) when there is no effective biological control (Le Pelley and Subirana 1973). Work conducted by the National Center for Coffee Research (*Centro Nacional de Investigaciones de Café- Cenicafé*) in Colombia showed that approximately 10% of the coffee berries remain on the trees and on the ground after harvest (Chamorro et al. 1995).

After harvest, coffee berries of all growth stages fall to the ground; berries are also dropped by pickers during the harvest, but these are mainly overripe and dry berries. The CBB has a greater capacity to multiply in more developed berries, and in the face of adverse conditions (drought, high temperature), progeny can remain within the berry for several generations, becoming the main source of infestation of new crops (Cárdenas, 1991; Arcila et al. 1993; Peralta 1995; Bustillo et al. 1998). In addition, this insect is able to multiply in berries in the soil and then disperse (Castaño et al. 2005). The coffee berries that remain attached to the plants or that fall to the ground serve as shelter and food for CBB during the most critical period of its subsistence, which is the time from the end of harvest until the next crop reaches an adequate dry matter content to be bored (Ruiz 1996).

Given the conditions of the Colombian central coffee zone, it has been determined that at least two CBB generations can be produced from the moment that the fruit is susceptible to attack until the harvest season, but if berries are not harvested and become overripe or dry on the tree or fall to the ground, up to four or five generations can quickly reproduce under favorable environmental conditions (Ruiz 1996; Jaramillo 2011; Giraldo et al. 2018). In these berries, the CBB continues to reproduce for 5 months, and a single berry has the capacity to house 25 to 150 adults (Salazar et al. 1993). If coffee berries are not collected in a timely manner, then this factor greatly favors infestation during the next harvest season. In one coffee plantation that has been pruned and where a sanitation harvest was not performed, it is estimated that between 7 and 9 million beetles can be left on the ground for more than 140 days (Castaño et al., 2005). It has been calculated that approximately 1.7 million CBB flew per hectare in 2008 (La Niña), and up to 4.5 million flew in 2010 (El Niño) (Castaño et al. 2005; Benavides 2011).

In Colombia, the environmental conditions in the central coffee zone allow continuous fruit production that is divided into two harvest seasons, one during the first 6 months of the year and a main harvest in the second half. This contributes to the reproduction of the CBB by providing a source of food throughout the year (Bustillo 2008; Arcila 2011). Additionally, the metabolism of the insect is influenced by the external climate conditions such as temperature, humidity, irradiance, and precipitation. The thermal conditions necessary for the development of the CBB range between 13.9–15°C and 25–27°C is the optimal reproductive temperature (Jaramillo, 2011; Giraldo et al. 2018). At temperatures below or above this range, development and oviposition completely cease (Jaramillo et al. 2011). Temperature is the most important climatic variable that directly affects the biological development of insects and other living beings, affecting the number of generations per year, the distribution, the interaction with plants and natural enemies, and behavior (Bustillo, 2008; Deutsch et al. 2008; Thomson et al. 2010; Jaramillo et al. 2011; Giraldo et al. 2018).

Climate variability associated with the El Niño and La Niña phenomena alters the average annual air temperature. In Colombia, such variation is between 1°C and 2°C, while the variation in precipitation is between approximately 20% and 40%, which leads to extreme climate conditions in some regions of the country (Jaramillo 2005; Jaramillo and Arcila 2009b) that, in addition to the problems related to pests and diseases, have negatively impacted coffee production in recent years. Therefore, the present study was conducted under field conditions to estimate the effect that CBB-infested ground berries have on coffee tree infestation across different elevations and climatic conditions.

## Materials and methods

The study was conducted in the Cauca-Colombia River basin from November 2007 to August 2010. Four sites were chosen at 1,200, 1.390, 1450, and 1,700 masl (Table 1).

**Table 1:** Study locations and climate conditions (historical averages) recorded at the Cenicafé weather stations near the study areas

Location—department	Altitude (m)	Average temperature (°C)	RH (%)	Solar irradiance (h)	Precipitation (mm)
Paraguaicito-Quindío	1.218	21.7	78	1,747	2,161
Naranjal-FMM-Caldas	1,381	20.9	78	1,763	2,782
Finca La Bella-Quindío	1,470	20.4	79	1,420	2,176
Finca Sta Cruz-Risaralda	1,700	19.4	81	1,488	2,662

### Effect of bored berries on the ground on the infestation of the tree

**Fig. 1:**
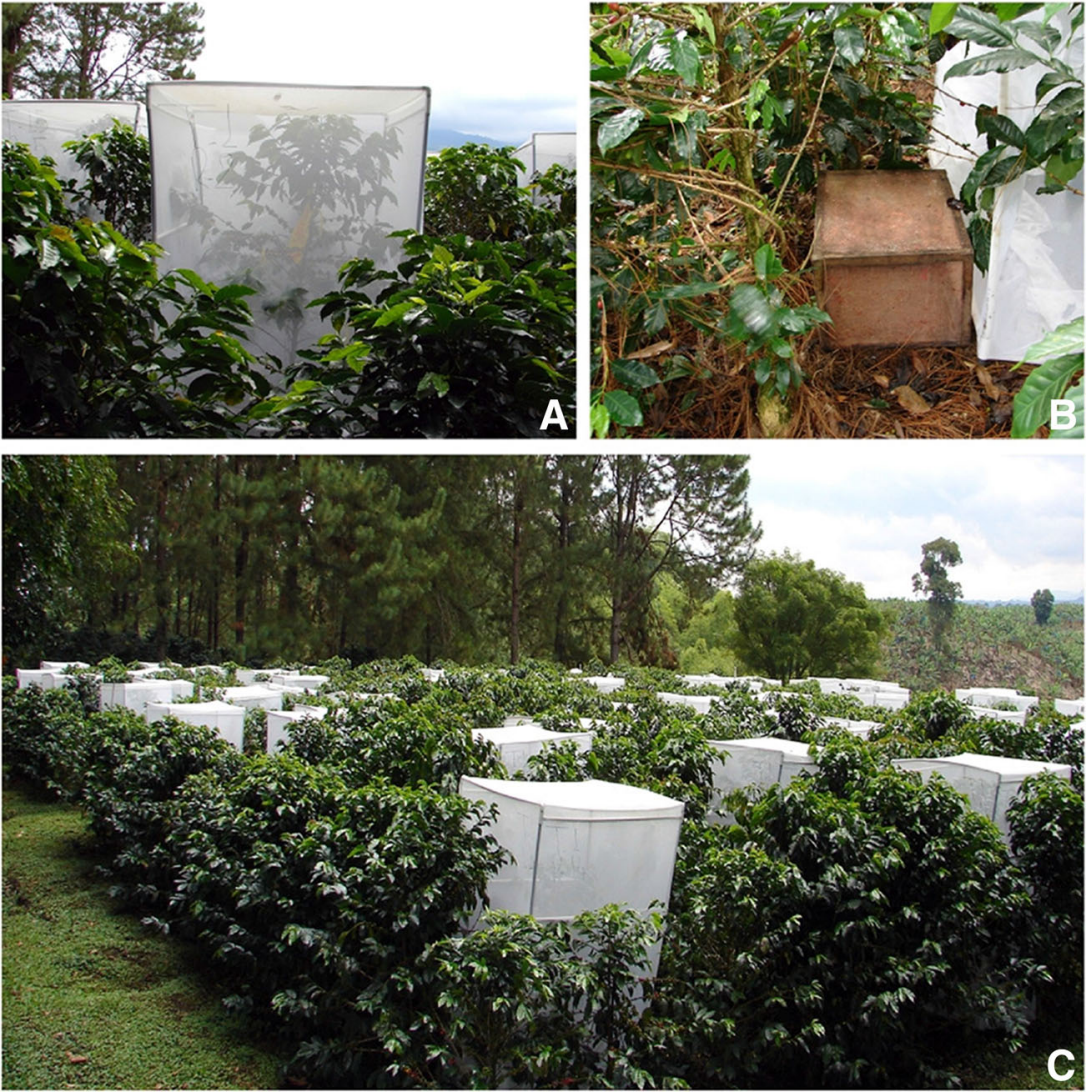
Experimental plot. (A) Sampling unit (coffee tree covered with entomological cage) to determine infestation of coffee berry borer (CBB) *Hypothenemus hampei* on the tree from emerged adults from bored berries placed on the ground. (B) Sticky cage trap next to the sampling unit to evaluate emergence of CBB adults. (C) experimental plot with 6 treatments: (0, 1, 5, 10, 15, 20) bored berries placed on the ground in each sampling unit with 15 replicates

At each site, a plot of 5,000 trees of *Coffea arabica* in the third harvest of the season was identified. In each plot, 90 coffee trees with 120–150-day-old developing fruits were randomly selected for the study. In the experimental plots, no insect control was conducted during the study. The sampling unit consisted of a tree covered with an entomological cage (2.10 m H × 1.30 m W × 1.30 m L) constructed of PVC pipes and lined with white muslin cloth (mesh size 0.1 mm; Fig. 1(A)). Prior to the study, the selected trees were verified as free of infested fruits on the trees and ground. The base under each tree was also cleared before the placement of infested berries. In each sampling unit, the treatments were randomly assigned to each tree and consisted of 1, 5, 10, 15, or 20 infested coffee berries placed on the ground under the tree (T1, T5, T10, T15, and T20, respectively). The absolute control consisted of a tree that was left uncovered and without fruits on the tree and ground (T0; Fig. 1(C)). For each of the five treatments and the control, 15 replicates were sampled in each plot. Infested berries were supplied by the Biocafe laboratory (Chinchina, Caldas). Each ripe berry (215 days post-flowering) was artificially infested with three fertile adult females 25 days prior to the study. Before placing berries on the ground, a random pool of 100 infested berries were dissected under a stereomicroscope to estimate the average number of CBB individuals per berry. From this, we estimated that 25 days after artificial infestation, the average number of CBB per berry was 7 ± 3.2 individuals (a mix of all stages: eggs, larvae, pupae, and adults). The evaluations were performed every 30 days for 6 months (productive cycle) over 4 years, during which time four climate conditions were considered: Neutral, La Nina, El Nino, and El Nino/La Nina transition. To know percent infestation for each tree, we recorded the total number of fruits on the tree and the number of infested fruits for each 30-day evaluation period. At the end of the 6 months, infested ground berries were dissected and the total number of CBB in each life stage was counted and recorded.

The research question and variable of interest was the number of CBB-infested berries in the soil and the total number of coffee berries that can be infested on the branches of the trees during four productive cycles. The climatic events and altitude were considered complementary variables.

### Adult CBB flight from bored berries on the ground

To evaluate the total number of CBB adults that fly from the berries on the ground, trees adjacent to each sampling unit for the previous activity were selected in each plot for a total of 90 trees per plot. At the base of each tree, we placed a 50 cm H × 40 cm W × 40 cm L trap cage (emergence trap) lined with muslin cloth which was saturated on the interior with Biotrampa® glue (Agrivar LTDA, Bogotá) (Fig. 1(B)). Each emergence trap cage was placed outside next to the sampling unit of each tree covered with an entomological cage. The treatments (T0, T1, T5, T10, T15, and T20) for each cage trap (sampling unit) consisted of 0, 1, 5, 10, 15, or 20 bored fruits placed on the ground, respectively, and were assigned as in the previous bioassay, corresponding to the six treatments replicated 15 times. Infested fruits were supplied by the Biocafé laboratory (Chinchiná, Caldas). Each ripe berry was artificially infested as explained above. In each cage trap with glue, the numbers of emerged (trapped) CBB adults were counted every 10 days for 6 months (productive cycle). After recording at each evaluation, the traps were cleaned and the trapped CBB adults were removed with a brush, and fresh glue was added again. At the end of the evaluation period, the fruits were dissected to account for the CBB stages that did not emerge. For each climate scenario (6-month period), new fresh infested dry fruits were placed on the ground in each sampling unit at the beginning of each experiment, for both the entomological cage covering the tree and the sticky cage trap. The cumulative number of CBB adults flying from the fruits on the ground was determined for each altitude and treatment by counting the number of adults captured in each sticky cage trap.

### CBB infesting unripe and uninfested berries on the ground

To evaluate infestation of unripe berries by the CBB on the ground, the same sampling units from the first activity were used, and 50 120-day-old, unripe berries were marked with indelible white ink to distinguish them from other berries and placed on the root vicinity of each tree next to the bored berries. The evaluations were conducted every 10 days for 6 months (180 days) over 4 years. The number of painted bored berries was recorded in each evaluation per sampling unit. At the end of the 6 months, infested ground berries were dissected and the total number of CBB in each life stage was counted and recorded.

### Number of bored berries on the tree and emergence response to climate variables

The meteorological stations were located in the four localities: Paraguaicito (Buenavista, Quindío, 1218 m), Naranjal (Chinchiná, Caldas, 1381 m), La Bella (Calarcá, Quindío, 1470 m), and El Jazmín (Santa Rosa de Cabal, Risaralda, 1700 m). In each locality, for each of the four different climate scenarios, the maximum, minimum, and average temperature (°C); solar irradiance (W/m^2^); relative humidity (RH, %); and precipitation (mm) were recorded daily and CBB development was compared across different localities.

### Data analysis

For each treatment, altitude, and climate scenario, the average number and the variation were estimated:

1. Average number of bored berries on the tree
2. Number of CBB that emerged from the bored berries on the ground
3. Average number of bored unripe berries on the ground

For each of the variables of interest, the mean and the variation expressed as ± standard error (SE) were estimated. Analysis of variance (ANOVA) was performed on the means and variation of each treatment and each evaluation in the completely randomized experimental design at the 5% level of significance using the statistical software SAS® v.9.0 (SAS Institute Inc., 2002). Significant differences were compared using a Dunnett test at the 5% level of significance to establish differences from the control treatment, and a correlation test was performed at the 5% level of significance to identify linear or nonlinear relationships between the variables of interest and the climate variables.

## Results

### Effect of bored berries on the ground on the infestation of the tree

**Fig. 2:**
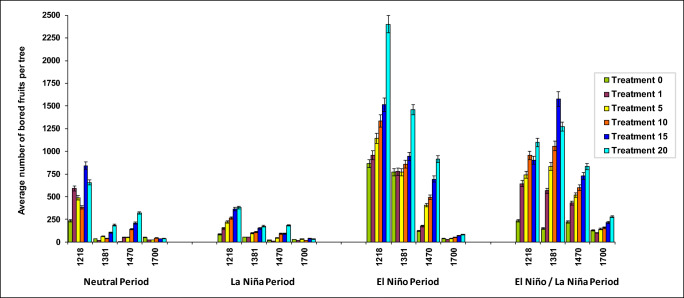
Average number of bored berries in trees at four altitudes under Neutral, La Niña, El Niño, and El Niño/La Niña transition climate scenarios (mean ± standard error, *n* = 15) over a period of 6 months for each treatment

The average number of bored berries per tree for each treatment, altitude, and climate scenario from day 30 to day 180 is presented in Table 2. Thus, at 1,218 m, a single bored berry on the ground (T1) resulted in significantly higher numbers of infested berries in the tree under the El Niño climate scenario (959 ± 89.6) compared to the Neutral scenario (590.2 ± 142.2) and La Niña scenario (151.5 ± 29.1) (*F* = 5.84, df = 5, *P* < 0.0001) (Fig. 2). In the locality at 1,381 m, a single bored berry on the ground (T1) resulted in significantly higher numbers of infested berries in the tree under the El Niño climate scenario (776.3 ± 117.2) compared to the Neutral scenario (16.6 ± 11.1) and La Niña scenario (54.8 ± 8.6) (*F*= 5.90, df = 5, *p* < 0.0001) (Fig. 2). In the locality at 1,470 m, a single bored berry on the ground (T1) resulted in significantly higher numbers of infested berries in the tree under El Niño climate scenario (176.0 ± 43.5) compared to the Neutral scenario (54.2 ± 13.2) and La Niña scenario (10.5 ± 2.3) (*F*= 5.04, df= 5, *p*<0.0001), but with much lower values of infested berries compared to lower altitudes (Fig. 2, Table 2). At 1,700 m, one bored berry on the ground (T1) did not show significant difference in the number of bored berries on the tree, with low values under the Neutral, La Niña, and El Niño scenarios, with low infestation values of 23.5 ± 4.9, 16.2 ± 4.0, and 29.3 ± 6.5 respectively (*F*= 13.58, df=2, *P* > 0.46) (Table 2 and Fig. 2). For the other treatments, the results are shown in Table 2 and Fig. 2.

**Table 2:** Number of bored berries per tree for each treatment, altitude and climate scenario (Neutral, La Niña, El Niño, and El Niño/La Niña transition climate scenarios) (mean ± standard error, *n* = 15) over a period of 6 months

Climate event	Treat.	Altitude			
		1,218 m	1,381 m	1,470 m	1,700 m
Neutral	0	234.1 ± 53.1 a	35.1 ±2.5 ac	6.0 ± 1.2 ac	51.3 ± 4.6 ac
La Niña	0	84.7 ± 8.7 a	54.5 ± 9.2 ac	20.9 ± 2.3 ac	30.2 ± 7.2 ac
El Niño	0	863.1 ± 121.4 b	769.4 ± 87.4 b	122.4 ± 14.2 a	39.0 ± 8.3 ac
El Niño/La Niña	0	234.0 ±34.1 a	150.2 ± 15.7 a	221.3 ± 33.2 a	130.8 ± 12.4 a
Neutral	1	590.2 ± 142.2 ab *	16.6 ± 11.1 ac	54.2 ± 13.2 ac*	23.5 ± 4.9 ac
La Niña	1	151.5 ± 29.1 a*	54.8 ± 8.6 ac	10.5 ± 2.3 ac	16.2 ± 4.0 ac
El Niño	1	959.0 ± 89.6 bc	776.3 ± 117.2 ab	176.0 ± 43.5 a	29.3 ± 6.5 ac
El Niño/La Niña	1	644.8 ± 97.4 ab	565.5 ± 42.6 ab*	430.6 ± 21.2 ab*	101,7 ± 8.6 a*
Neutral	5	488.5 ± 85.6 ab*	63.2 ± 16.1 ac*	51.0 ± 16.7 ac*	23.8 ± 7.1 ac*
La Niña	5	222.0 ± 34.5 a*	98.7 ± 45.6 ac*	47.5 ± 12.9 ac*	34.5 ± 8.0 ac
El Niño	5	1,142.8 ± 106.3 bc*	784.3 ± 75.3 b	407.9 ± 93.0 a*	38.5 ± 4.7 ac
El Niño/La Niña	5	744.1 ± 94.6 b*	834.4 ± 150.1	520 ± 30.4 ab*	144,2 ± 10.9 a
Neutral	10	379.9 ± 50.2 a*	41.1 ± 8.8 ac	140 ± 33.7 a*	44.7 ± 5.8 ac
La Niña	10	266 ± 38.3 a*	112.6 ± 35.4 a*	91.2 ± 17.8 ac*	31.4 ± 3.3 ac
El Niño	10	1,300 ± 103.6 bc*	863.5 ± 87.4 b	494.8 ± 47.5 ab*	53.2 ± 7.8 ac*
El Niño/La Niña	10	956 ± 99.7 bc*	1,051.4 ±129.1 bc*	598.3 ± 36.1 ab*	157.3 ± 11.1 a*
Neutral	15	842.6 ± 131.8 b*	102.3 ± 31.9 ac*	208.8 ± 42 a*	36.1 ± 4.3 ac
La Niña	15	365.7 ± 58.5 a*	150.4 ± 33.5 ac*	91.9 ± 15.1 ac*	40.7 ± 6.9 ac
El Niño	15	1,513.8 ± 116.3 bc*	944.1 ± 73.2 bc*	694.2 ± 77.6 ab*	71.3 ± 7.5 ac*
El Niño/La Niña	15	1,080.7 ± 116.6 bc	1,478.2 ± 113 bc*	731.6 ± 43.5 ab*	214.9 ± 20.6 a*
Neutral	20	657.3 ± 119.7 ab*	186.5 ± 50.5 a*	319.7 ± 73.3 a*	38.6 ± 7.3 ac*
La Niña	20	380.1± 62.5 a*	175.2 ± 31.7 a*	186.2 ± 29.1 a*	43.9 ± 7.2 ac
El Niño	20	2,336.8 ± 180.1 d*	1,458.9 ± 102.5 bc*	915.9 ± 97.5 bc*	83.7 ± 7.6 ac*
El Niño/La Niña	20	1,447.9 ± 134 bc*	1,537.8 ± 95.4 bc*	833.1 ± 40.4 bc*	266.3 ± 31.2 a*

*Average significantly different from the absolute control

Averages followed by the same letters indicate that there are no significant differences according to Dunnett’s test (*P* <0.05)

Over a period of 180 days at the end of the productive cycle, the mean number of bored berries on the tree was directly related to the number of bored berries on the ground, across varying altitudes and climate cycles (Fig. 2). Therefore, the minimum numbers of bored berries in the tree were observed under the treatment with one bored berry on the ground (T1) while the maximum values were observed in the treatment with 20 bored berries on the ground (T20). This value was intermediate in the other treatments (T1, T5, T10, and T15), exhibiting the same tendency to increase as the number of bored berries on the ground increased (Fig. 2). At 1,218 m, 20 bored berries on the ground (T20) resulted in significantly higher numbers of infested berries in the tree under El Niño climate scenario (2,330.8 ± 180.1) compared to the Neutral scenario (657.3 ± 114.7) and La Niña scenario (380.1 ± 62.5) (*F* = 5.9, df = 5, *P* < 0.0001) (Fig. 2).

At 1,381 m, 20 bored berries on the ground (T20) resulted in significantly higher numbers of infested berries in the tree under El Niño climate scenario (1,458.9 ± 102.5) compared to the Neutral scenario (186.5 ± 50.5) and La Niña scenario (175.2 ± 31.7) (*F* = 21.3, df = 5, *P* < 0.0001) (Fig. 2). At 1,470 m, 20 bored berries on the ground (T20) resulted in significantly higher number of infested berries in the tree under El Niño climate scenario (915.9 ± 97.5) compared to the Neutral scenario ( 319.7 ± 73.3) and La Niña scenario (186,2 ± 29.1) (*F* = 5.04, df = 5, *P* < 0.0001) (Fig. 2). At 1,700 m, 20 bored berries on the ground (T20) resulted in significantly higher number of infested berries in the tree under El Niño climate scenario (83.7 ± 7.6) compared to the Neutral scenario (38.6 ± 7.3) and La Niña scenario (43.9 ± 7.2) (*F* = 10.2, df = 5, *P* < 0.0001) (Fig. 2).

**Fig. 3:**
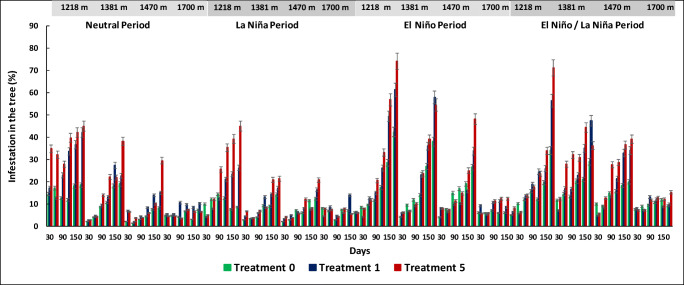
*Hypothenemus hampei* infestation percentages in tree berries per treatment (T0, T1, and T5), altitudinal range and average temperature under the Neutral, La Niña, El Niño, and El Niño-La Niña transition climate scenarios over a period of 6 months for each treatment

**Fig. 4:**
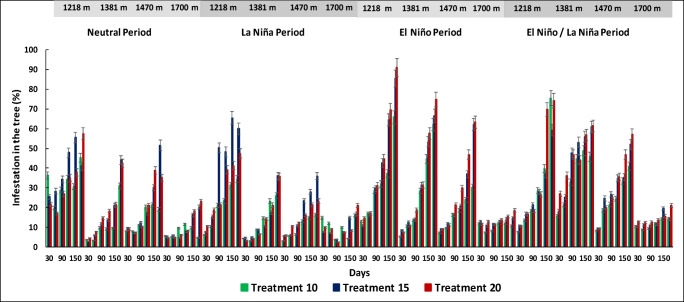
*Hypothenemus hampei* infestation percentages in tree berries per treatment (T10, T15, and T20), altitudinal range, and average temperature under the Neutral, La Niña, El Niño, and El Niño-La Niña transition climate scenarios over a period of 6 months for each treatment

We observed a significant increase in tree infestation from Neutral to El Niño period from day 30 to day 180 for each of the treatments relative to the control treatment and mainly occurred in Paraguaicito at 1,218 m, Naranjal at 1,381 m, and La Bella at 1,470 m (*F*=10.28, df=5, *P* < 0.05) but did not show significant difference in Santa Cruz at 1,700 m (*F*= 16.6, df=5, *P* > 0.46) (Figs. 3 and 4).

With 20 bored berries on the ground over a period of 180 days, the percentage of infestation of tree fruits was found to be greater at 1,218 m under El Niño climate scenario (91.6%) compared to the Neutral scenario (41.1%) and La Niña scenario (23.4%) (*F*= 13.3, *n*=15, df=5, *P*< 0.0001) (Fig. 4). At 1,381 m, the percentage of infestation of tree fruits was found to be greater under El Niño climate scenario (74.8%) compared to the Neutral scenario (36.9%) and La Niña scenario (23.3%) (*F*= 7.02, df=5, *P*< 0.0001). At 1,470 m, the percentage of infestation of tree fruits was found to be greater under El Niño climate scenario (77.6%) compared to the Neutral scenario (58.9%) and La Niña scenario (35.8%) (*F*= 28.1, df=5, *P*< 0.0001). At 1,700 m, the percentage of infestation of tree fruits did not show significant differences under El Niño climate scenario (18.4%) compared to the Neutral scenario (36.4%) and La Niña scenario (21.8%) (*F*= 0.65, df=5, *P*=0.6603) (Fig. 4).

**Fig. 5:**
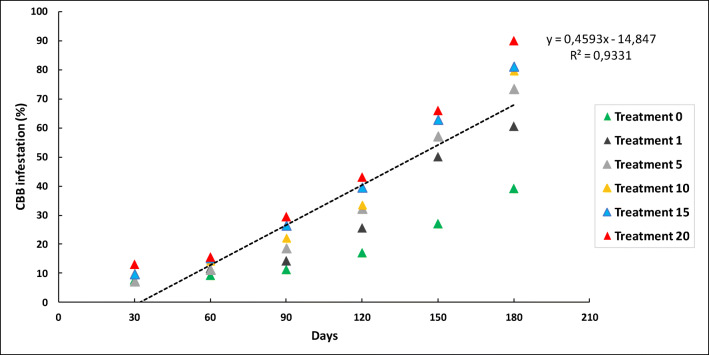
Relationship between the percentage of berries on the tree infested by *Hypothenemus hampei* over time per treatment during an El Niño climate event at an altitude of 1,218 m

The percentage of increase in the infestation in the fruits of the tree from day 30 to day 180 for each treatment during El Niño climate scenario is summarized in Fig. 5 With 1 bored berry on the ground (T1), the percentage of infestation in the fruits of the tree increases 4.2%, 12.7%, 15%, 26.1%, 49.4%, and 61.3% from days 30, 60, 90, 120, 150 and 180 respectively. The infestation percentages are lower when compared with the La Niña, a Neutral, and El Niño/La Niña transition periods for each treatment (number of bored berries on the ground) which can be observed in Figs. 3 and 4. During La Niña, the percentages of infestation in the tree were very low (< 3%) for all the altitudes, because the increase in soil moisture due to the winter causes the berries on the soil to rot faster, causing high mortality to the biological stages of the CBB, which is reflected in less infestation in trees and less adult emergence of the berries on the soil (Figs. 3, 4, and 8).

The relationship between the infestation of tree fruits and the time elapsed since treatment application was positive. At 1,218 m, we observed a significant difference in the mean number of infested tree berries under the neutral scenario for (T0); the regression function (*Y = 10.62 – 0.5269x*) indicated that the infestation on the tree was 8.5% at day 30 and 42.4% at day 180 at the end of the productive cycle. For the treatments T1, T5, T10, T15, and T20 at day 180, the infestation percentages on the tree branches reached 61.3%, 74.2%, 79.2%, 85.4%, and 91.1%, respectively.

The regression functions at 1,381 m (*Y = 15.607 – 0.467x*) and 1,470 m (*Y = 6.78 – 0.246x*) also showed a positive relationship between infestation and time. The infestation at day 180 were 38%, 57.8%, 54.5%, 62%, 66.6%, and 26.5% for treatments T0, T1, T5, T10, T15, and T20, respectively. At 1,470 m, the levels of infestation were 33.8%, 48.2%, 30.3%, and 62%, respectively, while no significant differences were observed between treatments in the locality at 1,700 m.

Under the El Niño climate scenario at an altitude of 1,218 m, the effect of the number of bored fruits on the ground on the level of infestation in the tree was adjusted to a quadratic linear regression (df = 4, *P* < 0.0001, *R*^2^ = 0.93). This same relationship was observed at 1,381 m (df = 4, *R*^2^ = 0.92) and 1,470 m (*F*=0.56; df = 4, *R*^2^ = 0.87) (Fig. 5).

### Adult CBB flight from bored berries on the ground

The peak in flight activity of adult CBB females emerging from infested fruits on the ground occurred during the first 30 days, followed by a gradual decrease up to day 140 (Fig. 9). However, the cumulative emergence of CBB adults asymptotically stabilized at day 80 of the evaluation (Fig. 9).

At an altitude of 1,218 m, from a single bored berry on the ground (T1), 1.3 CBB adults emerged in a Neutral period, 1.4 in a La Niña period, and 9.6 in an El Niño period. These values increased when 20 berries were left on the ground (T20); 6.1 CBB adults emerged in a Neutral period, 6.5 in a La Niña period, and 118.5 in an El Niño period.

For T20, at the localities at altitudes of 1,381 m, 1,470 m, and 1,700 m, a total of 78.2, 55.4, and 16.4 CBB adults emerged during an El Niño period, respectively, while those values were significantly reduced to 3.8, 5.3, and 6.1 adults, respectively, in a La Niña period (Fig. 8).

**Fig. 6:**
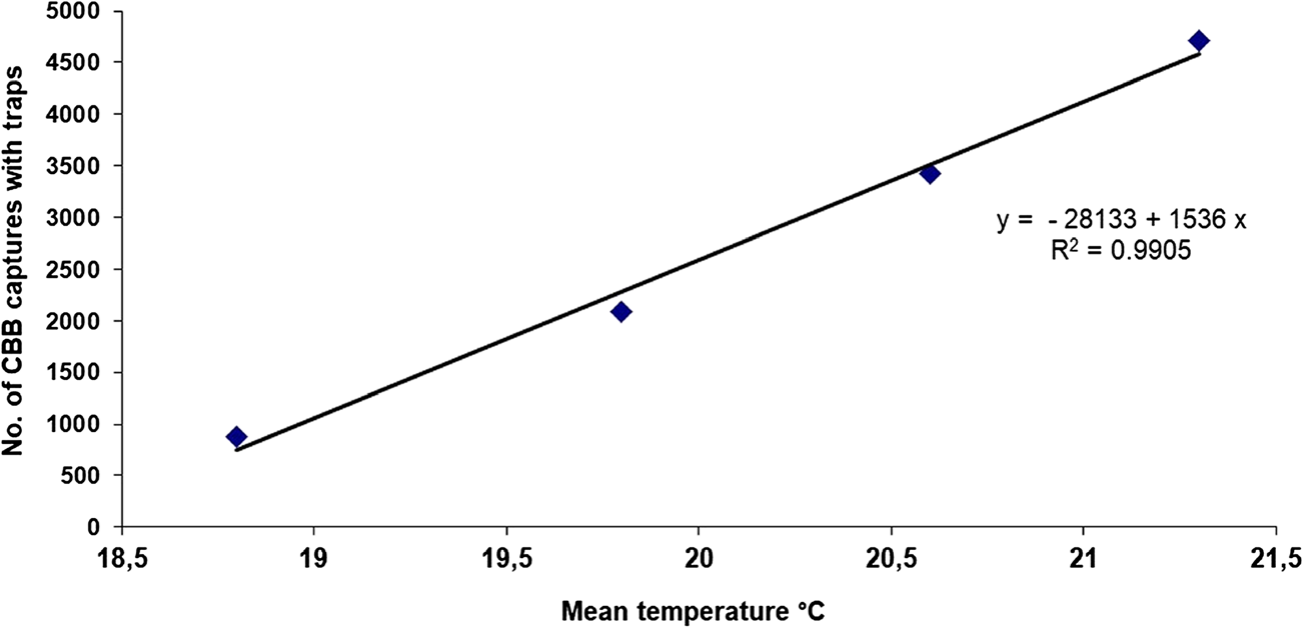
Positive correlation of adult CBB emergence from ground berries with temperature captured with traps

**Fig. 7:**
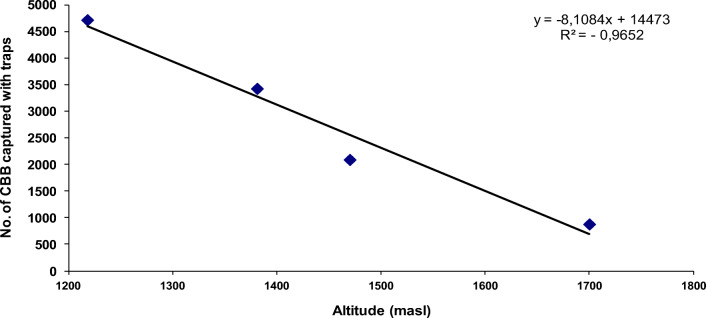
Negative correlation of adult CBB emergence from ground berries with altitude (m.a.s.l) captured with traps

**Fig. 8:**
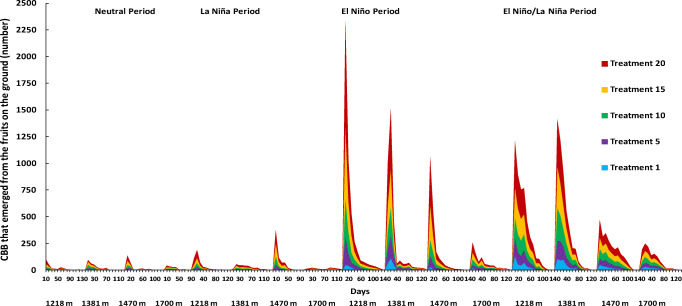
Number of adult CBB that emerged from berries on the ground by treatment at each altitude under the Neutral, La Niña, El Niño, and El Niño-La Niña transition climate scenarios

When counting the number of CBB adults that emerged by location and climate scenario, the peak in flight activity of adult CBB females emerging from infested fruits on the ground occurred during the El Niño period at the altitude of 1,218 m with a total of 4,717 ± 45.5 adults caught in the traps. Emergence decreased to 3,427 ± 23.3 adults in the locality at 1,381 m, to 2,091 ± 16.5 at 1,470 m, and to 879 ± 9.3 at 1,700 m (*F*= 3.12, df=5, *P*=0.0123, *P* < 0.05). Under a La Niña climate scenario, 461 ± 45 adults emerged at 1,218 m, 331 ± 53.4 at 1,381 m, 671 ± 62.2 at 1.470 m, and 186 ± 12.1 at 1,700 m (*F*= 3.12, df=5, *P* > 0.05) (Fig. 8). According to these results, the emergence of CBB adults was significantly positively correlated with temperature, with 879, 2091, 3427, and 4717 adults captured with traps at 18.8°C, 19.8°C, 20.6°C, and 21.3°C respectively (*F*= 3.2, df=5, *P*< 0.05, *R*^2^=0.99), while an inverse (negative) correlation was observed with altitude under the El Niño climate scenario at 1218 m, 1381 m, 1470 m, and 1700 m with the number of adults captured with traps (4717, 3427, 2091, and 879 adults respectively) (*F*=3.4, df *P* < 0.05, *R*^2^ = −0.96) (Figs. 6 and 7).

The results of Dunnet’s test revealed significant differences between treatments and altitudes and between an El Niño climate period and the Neutral and La Niña scenarios (Fig. 6). However, during the first productive cycle in a neutral period, the data exhibited high variability among treatments, and there were no statistically significant differences. This was because the bored berries used in the experiment were mature with live embryos, so many of them germinated, interrupting the borer beetle cycle of development and consequently diminishing the rate of adult flight. This problem was solved for the climate periods of La Niña 2008, El Niño 2009, and El Niño 2010, using mature dry coffee berries with dry embryos, which allowed the berries to last on the soil for 180 days and to serve as a refuge for the insects during the experiment.

**Fig. 9:**
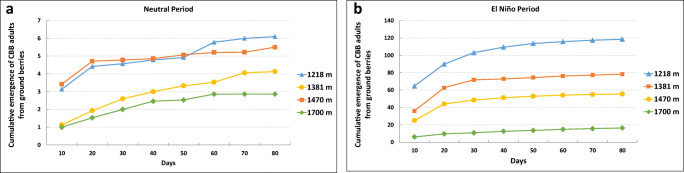
Cumulative emergence of adult CBB from 20 bored berries on the ground (*n* = 15) by altitudinal range during El Niño and Neutral climate events

This favored the percentage of CBB emergence with 99.4%, 48.6%, 40.8%, and 13.1% at 1,218 m, 1,381 m, 1,470 m, and 1,700 m, respectively, during an El Niño climate period and 22.3%, 5.7%, 19.5%, and 2% during a La Niña period (Fig. 8). This explains the marked increase in the CBB infestation percentages and the number of bored berries per tree during an El Niño period compared to a La Niña period, during which the level of infestation and the number of bored berries on the tree was lower (Fig. 9).

### Coffee berry borer infesting unripe and uninfested coffee berries

**Fig. 10:**
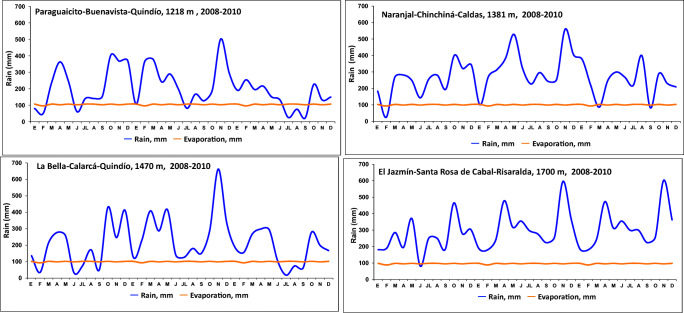
Precipitation and water balance in the experimental plots over time

Coffee berry borer infesting unripe and uninfested coffee berries placed at the base of the coffee tree in each sampling unit was significantly correlated with temperature and inversely correlated with altitude under the El Niño climate scenario (*n* = 15; *P* < 0.05; *R*^2^ = 0.96), reaching infestation values of 82%, 42%, 24%, and 5.6% at 1,218 m, 1,381 m, 1,470 m, and 1,700 m, respectively (Fig. 6). During a La Niña, the infestation levels did not exhibit a significant relationship with altitude (*n* = 15; *P* > 0.05; *R*^2^ = 0.46) in the localities at 1,218 m, 1,381 m, and 1,470 m, achieving infestation percentages of 27%, 29%, and 27% respectively. The infestation at 1,700 m went up to 5.2% (Fig. 10).

### CBB infestation rates and number of emerged adult response to climate variables

During the El Niño climate events in the lower-altitude localities (1218 m, 1381 m, and 1470 m) in this study, a prolonged drought that occurred in the months of June 2009 through February 2010 (Fig. 10) favored the emergence and flying of more CBB adults from the berries on the ground, as evidenced by sticky traps used to capture adult CBB, compared to during a La Niña climate period.

**Fig. 11:**
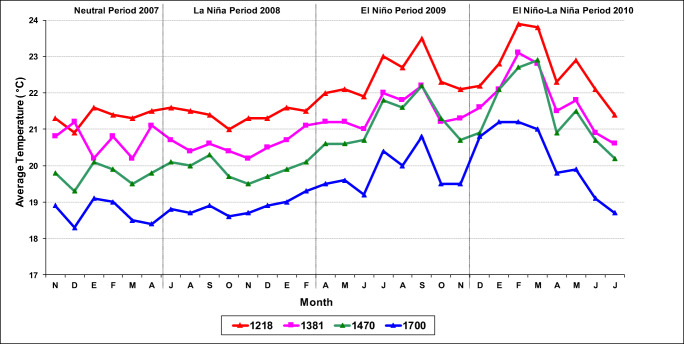
Variation in mean temperature over time during four different climate events in the four study locations (altitudes)

The questions and the working hypothesis of this work were fulfilled: the greater the amount of infested berries on the soil, the greater the infestation of coffee berries in the trees; with a higher environmental temperature, the levels of infestation of CBB in the tree were increased; during El Niño climatic event, with the increase in drought and environmental temperature (> 1–2°C), CBB levels increased and the flights of the insect were higher, and as a consequence of this, infestation and damage to the berries on the tree increased considerably (Figs. 2 and 11).

## Discussion

In the present study, we found that infestation of tree berries was positively correlated with the number of infested berries on the ground. In addition, we observed increased CBB emergence from ground berries and increased infestation in the trees during El Nino events (as a result of drought and an increase in air temperature of >1–2°C) compared to other climate scenarios.

The results of the present study demonstrated that the CBB is most problematic in localities below 1,300 m in altitude, where the levels of infestation are high due to average temperatures above 21°C at any time of the year and very high during El Niño climate events when temperatures reach 22°C or higher. In localities between 1,300 and 1,400 m with average temperatures of 20°C, the levels of infestation are intermediate to low during Neutral and La Niña periods. However, with temperatures of 21°C during El Niño periods, the infestation level is very high. In localities between 1,400 m and 1,600 m with temperatures of 19°C, infestation levels are low in Neutral and La Niña periods but intermediate in El Niño periods with temperatures of 20°C. In locations above 1,700 m with an average temperature of 18°C or lower, the insect presents no problems during any climate event or time of year. The previous results indicate the effect of climate conditions and altitude on the infestation rates and emergence of the CBB through variation in the average temperature and the thermal amplitude, namely, the difference between the maximum and minimum temperature, which varied between 9 and 13°C in the study locations. In terms of the average temperature, there was a difference between 1°C and 2°C above and below the monthly historical average temperature during the El Niño and La Niña climate events, respectively (Fig. 11).

These results demonstrate that CBB infestation rates and number of emerged adults are influenced by climate variables such as temperature, rainfall, solar irradiance, and precipitation during normal coffee growing conditions. Furthermore, the thermal regime is regulated by altitude, which allows average temperatures to vary within a range of 18 to 24°C that is favorable for coffee cultivation (Jaramillo and Arcila, 2009a; Jaramillo et al. 2009, 2011; Giraldo et al. 2018). Hamilton et al. 2019 studied CBB development across an elevational gradient on Hawai’i Island: applying laboratory degree-day predictions to natural field populations. Mean development time from egg to adult across all sites was 38.5 ± 3.46 days, while the mean time required for the completion of a full life cycle (from time of infestation to presence of mature F1 females) was 50.9 ± 3.35 days. This means that CBB development was faster at lower elevations as a result of higher daily temperatures, which is consistent with our findings. This translates to higher numbers of generations per season at low-elevation farms compared to high-elevation farms. This is important because it suggests that CBB management strategies will have to be adjusted to particular growing locations (e.g., low-elevation farms may have to spray *Beauveria bassiana* more frequently, and/or perform ground sanitation more thoroughly).

Johnson et al. (2019) studied postharvest population reservoirs of CBB in Hawaii and found that tree berries had significantly higher CBB abundance compared to both areas (ground below the tree foliage and ground between tree rows (20 vs 3–5 CBB per fruit)). Adult mortality was significantly higher on the ground (63–71%) compared to the trees (12%) and there was a positive correlation between ground raisin abundance and infestation in the trees. We found similar results, that ground raisin density was significantly positively correlated with tree infestation with higher populations of CBB on the tree, related to the abundance of ground infested berries.

The negative correlation of CBB emergence with altitude and positive with temperature obtained in this study can be explained by the study of Hamilton et al. (2019) that mention that the development time of CBB increases with increasing elevation and decreasing temperature with a mean requirement of 332 ± 14 degree-days and 386 ± 16 degree-days at 14.9°C and 13.9°C respectively. In other words, the thermal conditions necessary for the development of the CBB range between 13.9 and 15°C and 25–27°C is the optimal reproductive temperature (Jaramillo, 2011; Giraldo et al. 2018).

Due to the proximity of Colombia to the equator in the tropics, the average temperatures do not exhibit extreme variations during the year. However, climate events, such as El Niño and La Niña, can considerably increase or decrease the average temperature of the land as well as precipitation in some regions as a result of the warming or cooling of the Pacific Ocean, respectively.

In the Colombian coffee zone, the La Niña periods are characterized by an increase in rainfall and a decrease in the hours of solar irradiance and temperature, so the vegetative and reproductive growth and thus production of coffee plants is negatively affected (Jaramillo and Arcila 2009b). In contrast, the Earth’s climate is affected under El Niño scenarios as a consequence of the warming of the Pacific Ocean, with a decrease in rainfall in some regions and an increase in others that are associated with increases in solar irradiance and temperature. The Colombian coffee zone experiences an increase in the average temperature of between 1 and 2°C, and precipitation decreases by 20–40% throughout the Andean region (Jaramillo and Arcila 2009a) (Fig. 11).

**Fig. 12:**
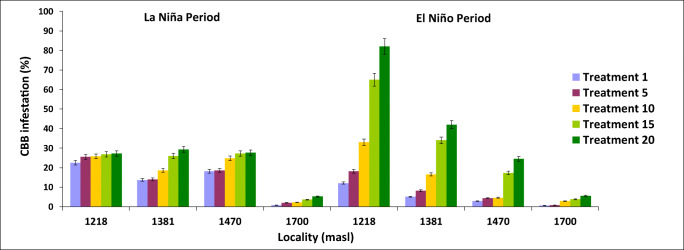
Percentage of berries on the ground infested by *Hypothenemus hampei* in relation to altitude, treatment, and climate scenario (mean ± standard error, *n* = 15)

The low emergence of CBB adults during La Nina events could be explained by increased mortality due to weather conditions (excess rainfall and high soil moisture content) that promote increased survival of natural enemies such as bacteria, nematodes, and *Beauveria bassiana*. These conditions may also promote the growth of saprophytic fungi which can accelerate the decomposition of berries on the soil, resulting in CBB mortality due to a lack of food (Ruiz 1996). In dry berries and during the El Niño climate periods, there was a marked water deficit and a decrease in soil moisture with the 1°C increase in average temperature in the months of January, February, and March 2009, which accelerated the rate of development and flying of CBB from the berries on the ground with the first rains after a prolonged drought period (Fig. 8 and 10). The number of CBB adults that emerged from the berries on the ground varied between treatments, altitudes, and climate scenarios (Fig. 12).

In terms of altitude, a difference of 0.7 to 0.8°C was evident for each 100-m interval, but during an El Niño climate event, the average temperature increased by between 1.0 and 1.5°C as altitude declined, as demonstrated in the four evaluated locations in this study (Fig. 11). These changes in temperature favor or limit the biology, development, and emergence of the CBB (Jaramillo et al. 2009, 2011; Mariño et al. 2016; Giraldo et al. 2018; Johnson et al. 2019; Hamilton et al. 2019). Therefore, in this study, there was a direct relationship between the infestation dynamics of the CBB and altitude with faster development of the insect in lower localities (1,218, 1,380, 1470 m.a.s.l) with average temperatures above 21°C. In contrast, CBB emergence is lower at sites above 1,600 m with average temperatures below 18°C (Figs. 6 and 7). These results agree with those reported by Castaño et al. (2005), who indicated that the greatest CBB flying came from unripe bored berries that remain on the ground in coffee plantations after pruning, and these berries were responsible for the dispersal of the insect to neighboring coffee plots when a sanitation harvest was not performed. This observation indicates that CBB adults remain alive for 140 ± 8.2 days in the berries on the ground, a value near 150 days, which is the maximum longevity reported for a CBB adult female. According to Castaño et al. (2005), the CBB infestation percentage in the neighboring coffee plots triples within the first 30 days. At 70 days after pruning, approximately 80% of the adult insect population has emerged, but flying can continue even after 150 days. This implies a constant flow of CBB to neighboring coffee plantations, which makes controlling this pest difficult and costly (Bustillo 2002; Castaño et al. 2005).

Although a direct relationship of increased infestation of CBB with precipitation and humidity was not observed through statistical analysis in this study, it could be descriptively observed that heavy rainfall stimulates CBB emergence and adult flights at the end of a prolonged drought period of more than two continuous months (Figs. 8 and 10).

Therefore, the CBB tolerates high temperatures and is favored by prolonged periods of water deficit during El Niño climate periods, which allow it to increase its rate of development and the number of generations per year, thus leading to higher levels of infestation and damage to coffee fruits (Jaramillo et al. 2011; Ramirez et al. 2014). Other studies under laboratory conditions have described the effects of increasing temperature on the development of the CBB (Jaramillo et al, 2010, 2011; Giraldo et al. 2018).

Increases in mean temperature are expected in the face of global climate change, which may cause changes in insect populations at different altitudinal ranges such as differences in insect-host plant interactions, alterations, and lags in the synchronization of host insect and parasitoid activity periods and changes to the growth and abundance, survival, feeding rates, and life cycles of herbivorous insects (Walther et al., 2002; Parmesan 2006; Menéndez 2007; Jaramillo et al. 2009; Hill et al. 2011). Therefore, knowledge of natural climate variation and the impacts of climate change on insects, both pest and beneficial, is important to prevent phytosanitary problems and to develop strategies to adapt to the expected changes.

The results of this study confirm the impact of bored berries on the ground on the infestation of a tree, although it varies with altitude and the climate conditions that occur over time in a given location. Based on the results of this study, it is confirmed that CBB can infest healthy, unripe berries on the ground after harvests and serve as a reservoir and refuge for the insect when there are no mature berries available on the tree, so it is important to collect the fallen berries from the soil to cut the insect reproduction cycle. As part of the integrated management of CBB, cultural control, timely harvesting practices, and the removal of ripe and unripe berries that have fallen to the ground or that remain on the plant are efficient and successful strategies to reduce pest populations in coffee crops and the level of infestation during the next harvest cycle (Bustillo et al., 1998).
